# Comparison of Heart Rate Variability Psychological Responses and Performance in Virtual and Real Archery

**DOI:** 10.1002/brb3.70070

**Published:** 2024-10-08

**Authors:** Nihal Dal, Serdar Tok, İlker Balıkçı, Said Enes Yılmaz, Erdal Binboğa

**Affiliations:** ^1^ Faculty of Sports Sciences Manisa Celal Bayar University Manisa Turkey; ^2^ Department of Biophysics Faculty of Medicine, Ege University Izmir Turkey

**Keywords:** heart rate variability, psychological responses, psychophysiology, vagal tone, virtual reality archery, virtual reality

## Abstract

**Background:**

This study examines the psychophysiological differences between virtual reality (VR) and real archery. It explores whether VR archery induces heart rate (HR), heart rate variability (HRV), and breathing rates similar to those experienced in real archery. Additionally, the study assesses differences in perceived anxiety, difficulty, confidence, rate of perceived exertion (RPE), and shooting performance between the two modalities, providing insights into the efficacy of VR as a training tool for archery.

**Methods:**

Twenty‐two (women: 8) individuals aged 20–24 participated in the study. We first recorded individuals’ resting HR, HRV, and breathing rates during baseline. Afterward, participants shot 10 real and virtual arrows from 18 m, whereas their HR, HRV, and breathing rate were measured, each lasting 4 min. Performance in VR and real archery was determined separately as the sum of the shots. We performed paired sample *t*‐tests to compare individuals’ performance, psychological, and psychophysiological responses recorded during VR and real arrow shooting. Afterward, we compared percentage changes between VR and real archery.

**Results:**

Results showed that HR and root mean square of successive differences (RMMSD) were significantly higher during real archery compared to virtual archery. In addition, VR archery led to a greater percentage change in RMSSD compared to real archery. Participants reported greater RPE and perceived difficulty after real archery. Performance was also higher during VR archery than real archery.

**Conclusions:**

Consequently, the results of the present study illustrated that VR, and real archery might lead to different autonomic response patterns in terms of vagal activity.

## Graphical abstract

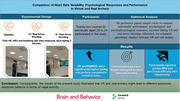

## 1 Introduction

Amidst the COVID‐19 pandemic, remote learning has become a necessity. Nonetheless, this presents a notable hurdle for physical education and sports instructors who aim to cultivate practical athleticism. Moreover, education and training in certain domains, such as aviation, the military, and athletics, can be costly and hazardous. Additionally, attaining peak performance in various fields, including athletics, necessitates supplementary training beyond conventional methods.

The sport of archery requires ample space and costly equipment to practice effectively. Even seasoned coaches and athletes can face challenges in teaching and honing technical and tactical skills. As such, innovative methods and strategies are necessary to instruct and train athletes in archery, especially when opportunities for training are limited, expensive, and potentially dangerous. Virtual reality (VR), which is described as a new technology that allows users to simulate a real object using a computer that can evoke a three‐dimensional (3D) atmosphere (Aprial, Purboyo, and Ansori 2019), might offer a solution to physical educators and coaches for teaching and training when physical training opportunities are limited in archery. VR technologies can convert certain real‐world experiences into virtual ones (Liu et al. 2020), which is well‐suited to teaching coordinated motor tasks (Oagaz, Schoun, and Choi 2022). VR aims to provide an immersive experience for its users. Immersion is a feeling where the user does not realize he/she is in an artificial world and is not aware of events in the outside world (Aprial, Purboyo, and Ansori 2019). Moreover, VR technologies might provide high ecological validity and experimental control for researchers (Loomis, Blascovich, and Beall 1999), especially in sports science.

On the basis of these possible advantages of VR, researchers examined whether certain technical and tactical skills in sports can effectively be simulated through VR technologies. For example, Bideau et al. (2003) found no difference in handball goalkeepers’ gestures facing real and virtual throwers, meaning that VR effectively simulates the interaction between goalkeepers and throwers in handball. A former study by Liu et al. (2020) found that a VR table tennis application can improve individuals’ performance in real‐world table tennis. Similarly, Oagaz, Schoun, and Choi (2022) also illustrated that VR‐based training might improve table tennis players’ technique and ball return ability. In addition to the abovecited studies, other studies demonstrated that VR applications can improve certain skills in soccer (Fortes et al. 2021) and golf (Harris et al. 2021).

As cited above, numerous studies have delved into the application of VR technology in sports like soccer, handball, golf, and table tennis. A few former research also examined the efficacy of VR‐based applications in enhancing technical proficiency in archery (Bedir and Erhan 2021; Yasumoto 2023; Zhang 2021). However, the question of whether utilizing a VR application for archery can elicit comparable autonomous nervous system (ANS) reactions to those produced during actual archery and consequently influence an archer's performance remains unanswered. As a result, well‐designed experiments must be conducted to delve deeper into this matter.

Engaging in a rigorous physical and mental activity like archery prompts ANS to elicit both psychological and physiological responses. The cardiovascular system is also regulated by the ANS via its sympathetic and parasympathetic branches. The sympathetic nervous system (SNS) results in increased heart rate (HR), narrowed blood vessels, and decreased gastrointestinal motility (Aubert, Seps, and Beckers 2003). Conversely, the parasympathetic nervous system (PNS) slows down the HR and is linked to the digestive system (Aubert, Seps, and Beckers 2003; Thayer and Lane 2009). Studies conducted previously have shown that during exercise, the activity of the PNS decreases, whereas the activity of the SNS increases (Burma et al. 2020; Rogers et al. 2021; White and Raven 2014). However, the opposite is observed during the recovery period, with an increase in parasympathetic activity (Martinmäki and Rusko 2008). The balance between the two systems is influenced by several factors, including the size of the left ventricle, the individual's fitness level, body posture, and mood state (Aubert, Seps, and Beckers 2003).

Heart rate variability (HRV) is a noninvasive method that can represent ANS activity as well as its regulation, which can be monitored through the calculation of the variation between normal‐to‐normal (NN) R–R interval in electrocardiogram (ECG) and arises from the interaction of the SNS and PNS branches of the ANS (European Task Force 1996; Heffernan et al. 2006; Miu, Heilman, and Miclea 2009). HRV is commonly analyzed in either the time‐domain or the frequency‐domain parameters. The most widely used time‐domain parameters are the standard deviation of NN intervals (SDNN), the root mean square of successive NN interval differences (RMSSD), and the mean NN intervals (NNmean) (Malik et al. 1996). RMSSD reflects vagal tone (Kleiger, Stein, and Bigger 2005; Thayer and Lane 2000), whereas SDNN represents both sympathetic and vagal activation of the ANS (Kleiger, Stein, and Bigger 2005). Frequency‐domain analysis focuses on the high‐frequency (HF) and low‐frequency (LF) powers of the variability changes corresponding to the activity of different branches of the ANS. LF power (0.04–0.15 Hz) is a marker of both cardiac sympathetic and parasympathetic activities (Dong 2016), whereas HF power (0.15–0.40 Hz) is an indicator of cardiac parasympathetic activity (Pagani et al. 1986). The low‐to‐high‐frequency (LF/HF) ratio determines the balance between sympathetic and parasympathetic activities (Reyes del Paso et al. 2013).

HRV is indicative of the efficiency of interaction among specific brain regions in the development of adaptive responses to environmental demands. Several HRV theories have been advanced to elucidate its capacity to document this interaction. As such, according to the neurovisceral integration model proposed by Benarroch (1993), the regulation of HR is controlled remotely by a network of brain areas known as the central autonomic network (CAN) to adapt to environmental demands. The CAN is composed of various regions of the brain, including cortical regions such as the medial prefrontal and insular cortices; limbic regions such as the anterior cingulate cortex, hypothalamus, and central nucleus of the amygdala; as well as brainstem regions such as the periaqueductal gray matter, ventrolateral medulla, and nucleus of the solitary tract. Therefore, as stated by Thayer and Lane (2000), the output of the CAN is directly linked to HRV.

In addition to the neurovisceral integration model, it is essential to note that other models concerning HRV also hold significance. For example, the biological behavioral model (Grossman and Taylor 2007) highlights the significance of vagal tone in regulating energy exchange by synchronizing respiratory and cardiovascular processes during metabolic and behavioral changes. A higher resting vagal tone is viewed as advantageous as it indicates a functional energy reserve capacity from which the organism can draw during more active states. In the same vein, the polyvagal theory proposed by Porges (2007) suggests that a greater vagal tone is connected to enhance social functioning. As per Laborde, Mosley, and Thayer (2017), a range of theories pertaining to HRV converge on the concept of vagal tone, representing a focal point within HRV research.

RMSSD, as an HRV time‐domain index, is assumed to effectively capture vagally mediated changes in HRV (Shaffer and Ginsberg 2017). Notably, Kleiger, Stein, and Bigger (2005) and Laborde, Mosley, and Thayer (2017) have underscored a pronounced correlation between RMSSD and HF, a frequency‐domain parameter of HRV reflective of vagal tone. Moreover, findings by Hill et al. (2009) have indicated that RMMSD is notably independent of respiratory influences. As a result of RMSSD's capacity to portray vagally mediated HRV, its application was favored for assessing distinctions in individuals’ responses to real and VR archery. Taken together, HRV, an essential index of central–peripheral neural feedback and the central nervous system (CNS) integration (CNS–ANS), might explore whether VR‐based athletic applications, archery in this case, might produce autonomic responses comparable to real athletic activities.

It is currently unknown whether VR‐based athletic activities influence HRV because there is no scientific evidence to support this claim. However, previous studies have shown that other types of VR games, such as horror and first‐person shooting games, might impact both HRV and psychological responses. For example, Lemmens, Simon, and Sumter (2022) demonstrated that the VR versions of horror and first‐person shooting games may cause a stronger sense of presence and lower HRV compared to their TV game counterparts. On the basis of the previous research by Alghamdi et al. (2017), it has been found that even a non‐immersive VR experience can lead to a significant increase in SNS activity, as evidenced by the elevated HR and electrodermal responses. Moreover, it has been demonstrated that VR can create a stronger sense of situational presence, which triggers emotional reactions identical to those experienced in real situations (Alghamdi et al. 2017). Further, Malińska et al. (2015) reported an increase in SNS activity due to a handling task executed via VR, as evidenced by an increase in LF power and LF/HF ratio. Finally, Jang et al. (2002) tested individuals’ HRV activity during flight and driving simulations and found that although VR flying simulation led to almost no changes in terms of HR and HRV indices, VR driving simulation gave rise to a significant HR acceleration and significant changes in HRV indices.

VR applications can influence physiological activity through various mechanisms. Previous studies (Himi et al. 2004; Malińska et al. 2015; Ohyama et al. 2007) have indicated that the conflicting visual and vestibular stimuli induced by VR, along with resulting motion sickness, can potentially impact HRV. However, as archery involves maintaining a stable body position and does not entail sudden, unpredictable direction changes, which is less likely to cause visual–vestibular conflict. Therefore, despite the possibility of visual–vestibular conflict due to VR environment, we expected that VR and real archery might produce similar HRV responses.

Numerous studies have explored HRV in sports and exercise settings, revealing that HRV parameters may indicate how athletes respond to physical and psychological stress (Makivić, Nikić Djordjević, and Willis 2013; Plews et al. 2013). HRV could also be linked to pre‐competition anxiety in athletes (Blásquez, Font, and Ortís 2009). Furthermore, research has indicated that HRV indices could serve as a valuable indicator of an athlete's recovery following an intense workout (Martinmäki and Rusko 2008; Plews et al. 2013; Vesterinen et al. 2016). Previous studies have also shown that HRV is a crucial parameter that can reflect archers’ psychophysiological responses during shooting and subsequent recovery (Tok et al. 2020). Moreover, Carrillo et al. (2011) have identified HRV responses associated with optimal performance in elite archers.

The above‐cited studies provide promising results that VR might provide an opportunity for skill learning and additional training for athletes. However, no previous study tested whether self‐report psychological and objectively measured psychophysiological responses caused by real and VR athletic activity, archery in this case, are comparable. Hence, it is not clear whether a VR‐based archery application might represent real archery effectively remains unknown and deserves careful examination. This study might present an opportunity to gain insights for athletes and coaches interested in ascertaining the potential for VR‐based athletic applications to enhance psychomotor skills, particularly in situations where access to formal training is constrained. Furthermore, this research could contribute to a deeper understanding of the interplay between the CAN and CNS in VR settings, which could be of great interest to researchers in this field.

In the present study, we aimed to explore whether psychophysiological activity represented by HR, HRV, and breathing rates might be comparable between VR and real archery. In addition, the secondary purpose of the study was to compare anxiety, confidence, difficulty, rate of perceived exhaustion (RPE), and shooting scores between virtual and real archery. Due to the abovementioned technical reasons associated with archery such as stable body position during arrow shooting, we predicted that both VR and real archery should give rise to similar HRV responses in terms of time (SDNN, RMSSD, NN50, and PNN50) and frequency (LF, HF, and LF/HF ratio) domain parameters. Moreover, there should not be any significant differences in terms of anxiety, confidence, difficulty, RPE, and shooting score.

## 2 Methods

### 2.1 Participants

Twenty‐two undergraduate students without previous experience in archery (8 females and 14 males) ranging in age from 20 to 24 (*M* = 21.7, SD = 1.8) participated in the study. Participants were enrolled in and completed a 14‐week archery class. Participants received extra course credits for their participation. Participants had no diagnosis of acute or chronic neuromuscular disease or psychiatric disorders. Participants were required to abstain from using medications affecting nervous system functioning for the duration of the study. The mean height and weight of the participants were 167.4 cm (SD = 1.8) and 71 kg (SD = 4.23), respectively. The local ethics committee approved all experimental procedures, participants signed informed consent before the study, and all data were collected following the latest version of the Helsinki Declaration.

### 2.2 Experimental Procedures

**FIGURE 1:**
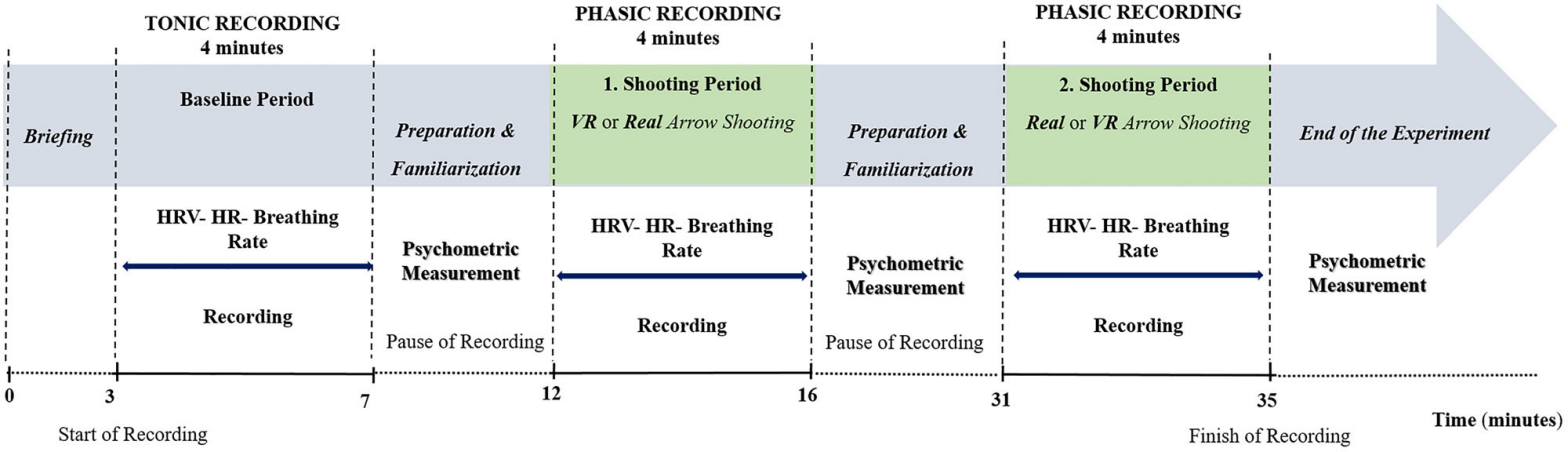
Schematic representation of the experimental procedure. The experiment involved three periods, each lasting for 4 min. In the first period, participants’ resting heart rate (HR), heart rate variability (HRV), and breathing rate were measured. Before each shooting periods, the participants were allowed to warm up, adjust their sight, and get familiar with the equipment in both virtual reality (VR) and real archery. Each individual reported their anxiety level and confidence just before each shooting session. Participants also self‐reported perceived difficulty and rate of perceived exertion (RPE) at the end of each shooting period. The order of the shooting sessions, either virtual or real, was randomized between the participants.

**FIGURE 2:**
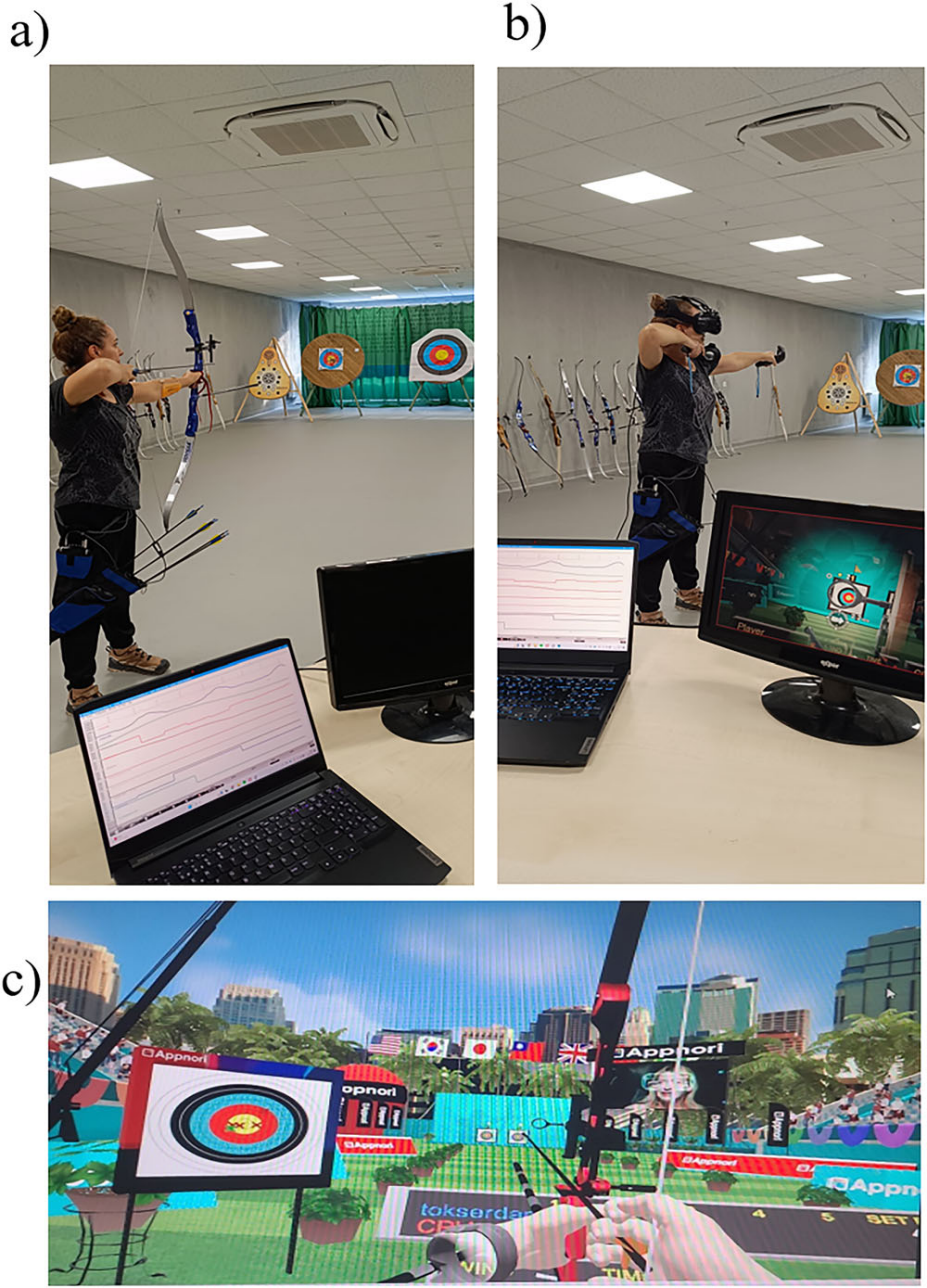
Experimental setups of real, virtual arrow shooting, and a screenshot of the Archery King virtual reality (VR) application. Note that part (a) shows the real arrow shooting during the experiment; part (b) demonstrates VR arrow shooting during the experiment via head‐mounted VR equipment, and part (c) illustrates a screenshot of the Archery King VR application.

HRV parameters can differ significantly from person to person, and for that reason, comparing groups can be challenging. To tackle this issue, we adopted a within‐subject repeated measures design to analyze the HRV responses of the same participants during baseline, VR, and real archery. We recorded baseline physiological activity in accordance with the recommendations of Laborde, Mosley, and Thayer (2017) and Quintana and Heathers (2014). As illustrated in Figure 1, the experiment consisted of three periods, lasting 4 min each. In the first period of the experiment, we recorded participants’ baseline HR, HRV responses, and breathing rates. Before the experiment, participants were allowed to warm up, adjust their bow site, or familiarization with VR equipment. Before each shooting session (virtual/real), individuals also reported their state anxiety and confidence level via a 10 cm visual analog scale (VAS). Afterward, participants shot 10 real and 10 virtual arrows from 18 meters to an 80 cm diameter target, whereas their HRV and breathing rate were recorded. Participants were allowed to rest and change equipment, adjust bow sight or put on VR equipment (head‐mounted display), and adjust joysticks for 15 min between VR and real archery sessions. Immediately after each shooting session, participants rated their perceived difficulty and RPE through 10 cm VAS. The order of the conditions (virtual or real) was counterbalanced between participants. Participants were given additional arrows if they finished the real shootings in less than 4 min. But only the first 10 shots were recorded, as the performance was determined as the sum of the first 10 shots. Moreover, during the virtual shootings, there was another virtual archer or non‐player character (NPC) in the virtual environment. The participants were able to see NPC if they turn the right‐hand side. However, the participants were instructed to ignore the NPC shooters and focus on their shots. The shooting task was self‐paced, so participants decided when to shoot an arrow and how long to prepare the shot. On the basis of a pilot study of five novice archers, we determined that participants were required to shoot one real or virtual arrow in approximately 20–25 s (Figure 2a,b). As depicted in Figure 2a,b, the participants were specifically instructed to employ identical shooting techniques in VR and real archery settings to reduce any disparities in the physical demands experienced during the shooting task. All experiments were carried out between 12 noon and 3 p.m. during the archery final 2021–2022 academic year exam at the end of the 14‐week course. Hence, participants had to accumulate 60 points to be rewarded with course credits. Participants were instructed not to consume foods or beverages, such as tea, coffee, or energy drinks, 2 h and alcoholic drinks 24 h before the experiment as they could influence HR and HRV. All participants were tested by the first researcher/author of this present study, who is also a former top‐class international archer. No audiences were present during the execution of the experiments. Participants were allowed to take six shots for familiarization and to adjust the bow's site.

### 2.3 Measures

#### 2.3.1 HR, HRV, and Breathing Rate

All physiological data were collected continuously during the experiment using a portable Nexus‐10 Mark II recording (Mind Media CV; Roermond, Herten, the Netherlands) and its software (BioTrace, Mind Media BV, the Netherlands) system as well as its accompanying sensors, which were connected to the recording computer via Bluetooth. HR and HRV data were recorded via ECG in a lead II configuration using three Ag/AgCl electrodes. One electrode was fixed below the right clavicle and one on the left side of the chest below the sixth rib. The ground electrode was placed under the left clavicle. ECG signals were saved at 24‐bit resolution with a sampling rate of 1024 Hz (50 Hz notch filter). On the other hand, breathing rates were measured with a NeXus respiration sensor using a magnet with a hall sensor (magnetic field sensor) to measure the stretching of the chest band. The chest band was placed on the abdomen at the umbilical level, providing the abdominal motion signals. The measured value is the raw data from the sensor and is related to the expansion of the chest belt. The breathing rate (calculated by the software in breaths per minute) is derived from the raw data.

#### 2.3.2 Perceived Level of Anxiety, Confidence, Difficulty, RPE, Shooting Score

To measure perceived state anxiety and confidence immediately before the VR and real arrow shootings, we used a 10‐cm VAS. The VAS was also implemented to assess RPE and perceived difficulty immediately after each shooting session (virtual/real). Participants’ performance was determined by the sum of the first 10 shooting scores during VR and real archery.

#### 2.3.3 Psychophysiological Analysis

All psychophysiological data were analyzed via Nexus‐10 Mark II recording (Mind Media CV; Roermond, Herten, the Netherlands) and its supplied software (BioTrace, Mind Media BV, the Netherlands). The HRV of each participant was obtained on the basis of the time series of peak‐to‐peak intervals that were immediately extracted from the ECG data. Before analyses, the peak‐to‐peak intervals were visually inspected for artifact rejection and unusual heartbeats (ectopic signals, premature signals), and then the corrected intervals were converted into the inter‐beat interval (IBI) time series. Following the recommendations of the European Task Force (1996), the HRV time and frequency‐domain analyses were performed by using the IBI time series. In the present study, we employed NNmean (ms), SDNN (ms), and RMSSD (ms) as the time‐domain indices. The frequency‐domain indices were computed via the FFT algorithm (FFT window length = 512, Hanning Window, 1024 points). In the present study, the power‐spectrum density was measured for the primary frequency indices: the LF and HF power (ms^2^). The relationship between these bands (LF/HF ratio) was also analyzed.

#### 2.3.4 VR Equipment and VR Archery Application

As demonstrated in Figure 2c, we used the *Archery Kings VR* gaming application (Appnori Inc., Busan, Republic of Korea, 2018) for the execution of the VR archery task, which is available in the Steam Library. The application was launched with an Nvidia GeForce RTX 2070 graphics card, 16 GB of RAM, and an AMD Ryzen 52600X six‐core processor desktop computer. The virtual environment was presented via HTC Vive Pro head‐mounted display (HTC Inc., Taoyuan City, Taiwan) with a resolution of 2880 × 1600 pixels that was updated 90 times/s and has a horizontal and vertical field of view of 110°. The VR apparatus gives the impression of a complete immersion in a virtual environment.

### 2.4 Statistical Analysis

We first log‐transformed (log‐10) all HRV frequency‐domain parameters before statistical analysis to satisfy the requirements of linear statistical analysis. Then, we performed a series of paired sample *t*‐tests to explore whether HR, breathing rate, and HRV responses differed significantly between VR and real arrow shooting. We also compared perceived anxiety, confidence, exertion, and performance between VR and real arrow shootings through paired sample *t*‐tests. Finally, we decided to normalize and express the physiological data recorded during both VR and real archery concerning a reference value. For this reason, we used physiological data recorded during baseline conditions as a normalization reference and defined, for example, as 100% HR. Then, psychophysiological data recorded during VR and real archery were divided by this reference and multiplied by 100. Therefore, we were able to express each physiological data as a percentage of its reference value (baseline level). In other words, we calculated how much physiological data recorded during both archery conditions deviated from the baseline level. Afterward, we compared these normalized physiological variables between VR and real archery through paired sample *t*‐tests. The level of significance was set to < 0.05. The data obtained from the present study were analyzed via IBM SPSS 21.

## 3 Results

### 3.1 HR, HRV, and Breathing Rate

**FIGURE 3:**
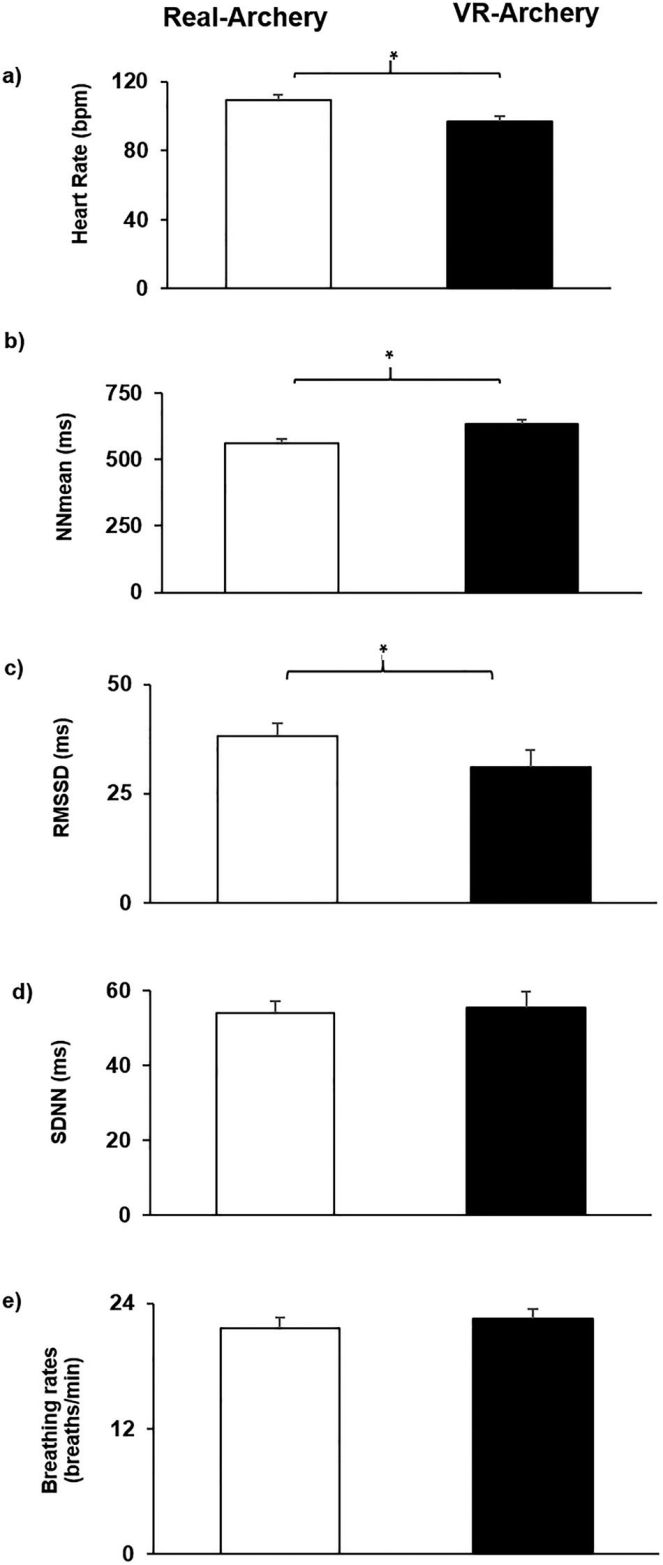
Heart rate (HR) (a), mean NN intervals (NNmean) (b), root mean square of successive differences (RMSSD) (c), standard deviations of NN intervals (SDNN) (d), and breathing rate (e) changes during virtual reality (VR) and real archery shooting trials.There were significant differences in the values of HR, NNmean, and RMSSD between the VR and real archery shooting trials. Specifically, HR and RMSSD values were notably higher during real archery compared to VR archery, whereas the NNmean value was significantly lower during real archery. It's worth noting that no significant differences were observed in SDNN and breathing rate between VR and real archery. *: *p* < 0.05.

To compare HR, HRV, and breathing rate differences between VR and real archery, we conducted a series of paired sample *t*‐tests. As illustrated in Table 1, HR measured during real arrow shooting was significantly greater than during VR arrow shooting. HRV time‐domain parameter of NNmean in real arrow shooting was significantly lower than in VR arrow shooting. On the contrary, the results revealed that the HRV time‐domain parameter of RMSSD in VR arrow shooting was significantly lower than RMSSD in real arrow shooting. Further, the log LF/HF ratio measured during the VR arrow shooting was significantly higher than the log LF/HF ratio measured during the real arrow shooting. Breathing rate, SDNN, log LF, and log HF did not differ between VR and real arrow shooting. Figure 3 further demonstrates the differences in terms of psychophysiological data recorded during VR and real archery.

**TABLE 1:** Comparisons of HR, HRV, and breathing rate during VR and real archery.

	Real archery		Virtual reality archery				
Variables	Means	SD	Means	SD	*t*	*p*	*d*
**Breathing rate (breaths/min [BPM])**	21.64	4.65	22.57	4.10	− 0.98	0.33	0.15
**HR (BPM)**	109.13	14.50	96.96	14.56	9.05	**0.00** ^*^	**0.95**
**NNmean (ms)**	560.39	71.09	631.29	79.09	− 9.86	**0.00** ^*^	**0.98**
**SDNN (ms)**	54.02	14.98	55.58	18.54	− 0.52	0.60	0.06
**RMSSD (ms)**	38.15	13.49	30.94	18.25	2.16	**0.04** ^*^	**0.43**
**Log LF (ms^2^)**	3.51	0.42	3.57	0.40	− 0.77	0.44	1
**Log HF (ms^2^)**	3.02	0.39	2.87	0.37	1.43	0.16	0.42
**Log LF/HF**	0.48	0.34	0.70	0.42	− 2.54	**0.01** ^*^	**0.72**

Abbreviations: HF, high‐frequency power; HR, heart rate; LF, low‐frequency power; LF/HF, ratio of low‐frequency power to high‐frequency power; NNmean, mean normal‐to‐normal (NN) HR intervals; RMSSD, root mean square of successive NN interval differences; SD, standard deviation;SDNN, standard deviation of NN intervals.

^*^ Results in bold were considered statistically significant if *p* ≤ 0.05.

**FIGURE 4:**
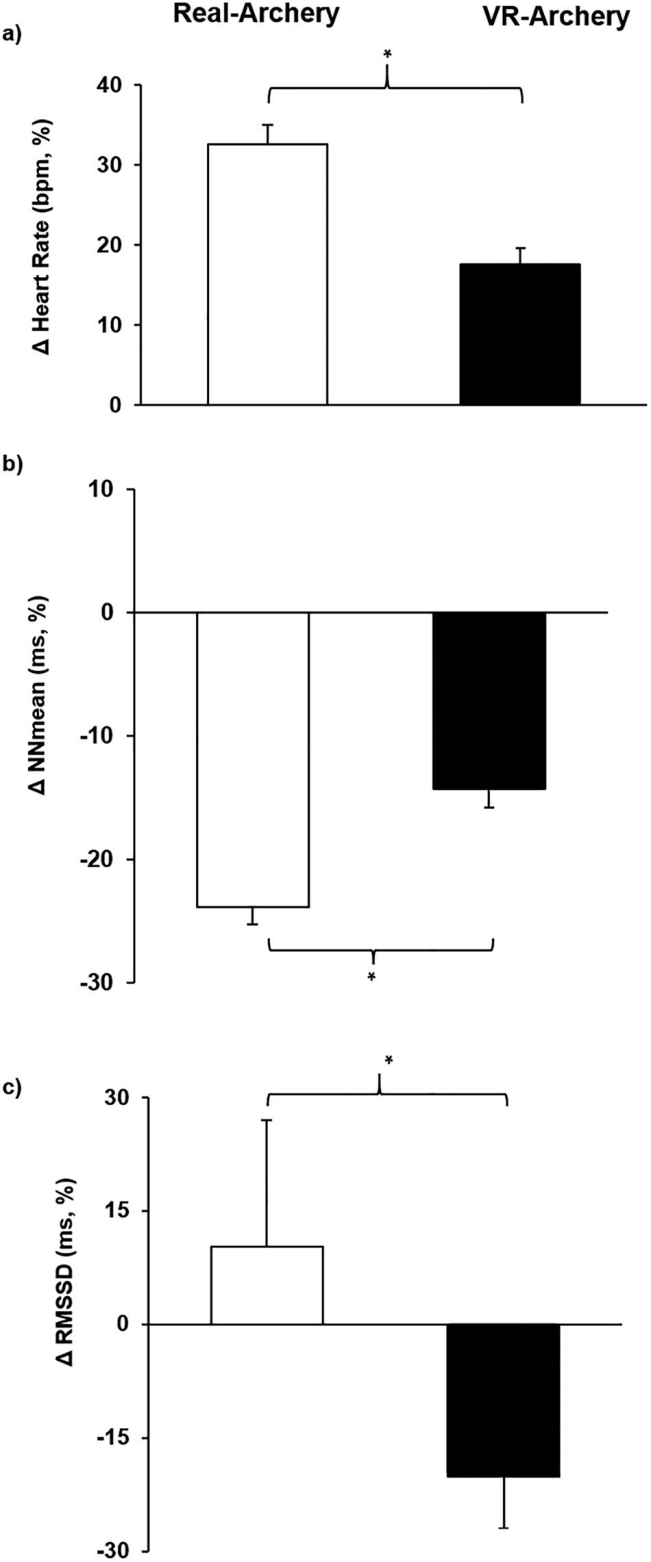
Percentage change of heart rate (HR), mean NN intervals (NNmean), and root mean square of successive differences (RMSSD) from baseline to virtual reality (VR) and real archery. Note that part (a) real archery resulted in a 32.50% increase in HR, whereas VR archery only led to a 17.54% increase; (b) real archery decreased NNmean by 23.83%, whereas VR archery only decreased it by 14.26%; and (c) real archery resulted in a 10.26% increase in RMSSD, whereas VR archery led to a decrease of 19.77% in RMSSD. *: *p* < 0.05.

As illustrated in Table 2, paired sample *t*‐tests showed that the percentage change of HR measured during real arrow shooting was significantly greater than during VR arrow shooting. Similarly, the results revealed that the percentage change of NNmean in VR arrow shooting was significantly lower than the percentage change of NNmean in real arrow shooting. Further, the percentage change of RMSSD in VR arrow shooting was significantly higher than the percentage change of RMSSD in real arrow shooting. Examination of descriptive statistics revealed that although there was a 10.26% RMSSD increase during real arrow shooting, there was a − 19.77% decrease during VR arrow shooting. Percentage changes of respiration rate, SDNN, log LF, log HF, and log LF/HF were not significantly different between VR and real shooting. Figure 4 illustrates the percentage changes of all psychophysiological data in relation to their baseline values.

**TABLE 2:** Comparisons of percentage change of HR, HRV, and breathing rate during VR and real archery in relation to its baseline values.

	Real archery (%)		Virtual reality archery (%)				
Variables	Means	SD	Means	SD	*t*	*p*	*d*
**ΔBreathing rate (breaths/min [BPM])**	39.06	66.99	45.55	72.04	− 1.05	0.30	0.07
**ΔHR (BPM)**	32.50	11.71	17.54	9.60	9.00	**0.00** ^*^	**1**
**ΔNNmean (ms)**	− 23.83	6.40	− 14.26	6.94	− 9.79	**0.00** ^*^	**1**
**ΔSDNN (ms)**	− 3.48	33.31	− 1.28	35.33	− 0.42	0.67	0.05
**ΔRMSSD (ms)**	10.26	76.78	− 19.77	37.08	2.39	**0.02** ^*^	**0.52**
**ΔLF (ms^2^)**	− 0.008	12.68	1.63	11.77	1.99	0.06	0.09
**ΔHF(ms^2^)**	− 1.40	21.52	− 7.34	15.02	1.79	0.08	0.28
**ΔLF/HF**	− 114.20	476.17	− 57.94	367.95	− 0.77	0.45	0.09

*Note*: Δ represents each variable's percentage change during shooting sessions in relation to its baseline values..

Abbreviations: ΔHR, heart rate; ΔHF, high‐frequency power; ΔLF, low‐frequency power; ΔLF/HF, the ratio of low‐frequency power to high‐frequency power; ΔNNmean, mean normal‐to‐normal (NN) HR intervals; ΔRMSSD, root mean square of successive NN interval differences; SD, standard deviation; ΔSDNN, the standard deviation of NN intervals.

^*^ Results in bold were considered statistically significant if *p* ≤ 0.05 difference from the baseline value.

### 3.2 Psychometric Results

**FIGURE 5:**
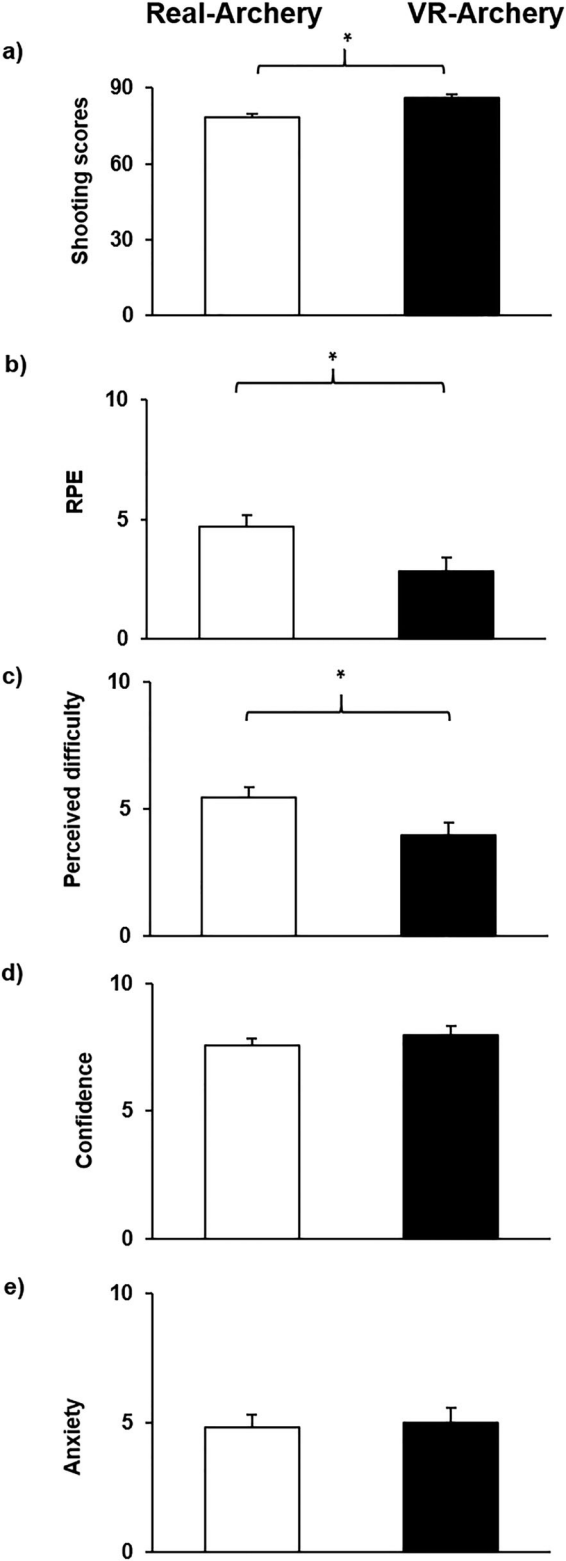
Differences in shooting score, rate of perceived exertion (RPE), perceived difficulty, state confidence, and anxiety. Note that (a) the participants achieved higher shooting scores during virtual reality (VR) archery as compared to real archery; (b) the participants reported a greater RPE during VR archery; (c) the level of perceived difficulty was higher during real archery as compared to VR archery, (d) there was no significant difference in the level of state confidence reported by individuals during VR and real archery; and (e) the difference in state anxiety was not statistically significant. *: *p* < 0.05.

As illustrated in Table 3, paired sample *t*‐tests yielded that the difficulty reported in real archery was significantly greater than in VR archery. Similarly, the rate of perceived exhaustion reported in real archery was significantly higher than in VR archery. Furthermore, the shooting score in VR archery was significantly higher than in real archery. Figure 5 shows differences in subjectively measured psychological data between VR and real archery.

**TABLE 3:** Comparisons of anxiety, confidence, perceived difficulty, RPE, and shooting scores during VR and real archery.

	Real archery		Virtual reality archery				
Variables	Means	SD	Means	SD	*t*	*P*	*d*
**Anxiety**	4.76	2.44	4.76	2.54	0.00	1.00	0.05
**Confidence**	7.62	1.28	7.90	1.70	− 0.795	0.436	0.12
**Perceived difficulty**	5.43	1.93	4.00	2.47	2.72	**0.01** ^*^	**0.81**
**RPE**	4.86	2.19	2.90	2.73	3.32	**0.00** ^*^	**0.93**
**Shooting score**	78.81	6.43	86.86	4.83	− 7.60	**0.00** ^*^	**1**

Abbreviations: RPE, rate of perceived exhaustion; SD, standard deviation.

^*^ Results in bold were considered statistically significant if *p* ≤ 0.05.

## 4 Discussion

The purpose of this study was to compare the psychophysiological responses of participants engaged in real and VR archery, as well as evaluate differences in anxiety, confidence, difficulty, RPE, and shooting scores. The results indicated that real archery provoked a greater increase in HR than VR archery, with a 32.5% increase observed in the former and a 17.5% increase in the latter. Specifically, real archery increased RMSSD by 10.2%, whereas VR archery reduced it by 19.8%. In addition to physiological parameters, certain perceived psychological responses also differed between VR and real archery. Notably, participants found real archery to be more challenging, with a higher RPE, in comparison to VR archery. Furthermore, participants produced a greater level of shooting accuracy (higher shooting scores) in VR archery than in real archery. On the contrary, there were no significant differences in terms of state anxiety and confidence parameters between real and VR archery.

In this study, a noteworthy discovery was made that warrants attention. Despite a significant rise in HR during real archery, an unexpected increase in RMSSD was observed. Existing literature suggests that heightened HR should cause a decrease in vagal activity (Appelhans and Luecken 2006; Aubert, Seps, and Beckers 2003; Berntson et al. 2005), yet the findings of this study indicate that vagal activity actually increased by 10% during real archery. Several theoretical perspectives could explain the augmented RMSSD observed during real archery. One possibility is the rebound effect, which is known to occur during recovery after an acute exercise session (Danieli et al. 2014; Stanley, Peake, and Buchheit 2013). In this instance, the SNS may suppress PNS activity, but it can also increase it (Delaney and Brodie 2000; Hall et al. 2004). The results of this study imply that the rebound effect may also arise during a physically demanding activity, such as real archery. Another factor that could account for the elevated RMSSD observed during real archery is the intervals between arrow shots. Archers have 10–12 s to prepare for the next shot, which enables them to cool down. During these intervals, archers regulate their breathing and reduce their arousal.

When compared to real archery, VR archery produced a notable − 19% reduction in RMSSD, whereas HR increased less significantly. The main reason for the vagal withdrawal evidenced by the RMSSD decrease might be visual–vestibular conflict. Spatial orientation is a complex process that relies on the brain's ability to simultaneously process visual, vestibular, and sensory inputs. However, head‐mounted VR devices can interfere with this process by providing conflicting stimuli to the brain. This can create a disconnect between the sensory patterns associated with real movement and the visual information received by the eyes (Himi et al. 2004; Malińska et al. 2015). The results of the previous studies also support our finding and argument indicating that a vagal withdrawal might occur during VR archery. For example, Ohyama et al. (2007) argued that VR systems can lead to incoherent visual–vestibular conflict, which eventually facilitates motion sickness. More importantly, Ohyama, Nishiike, and Watanabe (2007) demonstrated that visual–vestibular conflict induced by VR systems might lead to an increase in SNS activity. Similarly, Malińska et al. (2015) found that VR‐induced visual–vestibular conflict might lead to an increase in LF spectral power and LF/HF ratio, which means that VR application exposure can increase users’ SNS activity. Additionally, Himi et al. (2004) posited that symptoms associated with a heightened SNS may serve as a defensive response to the sensation of nausea. A recent investigation by Park, Ha, and Kim (2022) vestibular conflict could potentially cause changes in time‐domain indices of SDNN and PNN50. Although the current study did not specifically examine motion sickness, the VR exposure employed herein may have the potential to trigger it. Consequently, we have determined that the reduction in RMSSD observed during VR archery, which indicates vagal withdrawal, was principally attributable to visual–vestibular conflict, thereby providing a sound theoretical foundation for this conclusion. Other possible reasons might be the feeling of discomfort and stress due to head‐mounted VR devices (Malińska et al. 2015). In contrast to real archery where breaks can be taken to reduce tension, VR archers must remain vigilant to avoid accidents and falls. Moreover, the thrill, pleasure, and inquisitiveness associated with VR archery can potentially lower RMSSD levels.

We have observed distinct psychological variations between VR and real archery. To better comprehend these subjective differences, we can leverage the concept of fidelity—how accurately a simulation mirrors real‐world behavior and conditions. Scholars, such as Burdea and Coiffet (2003) and Gray (2019), have explored this framework, providing valuable insights into interpreting such distinctions. Table 3 shows no significant differences in anxiety and confidence levels before VR and real archery, suggesting that the VR task is psychologically and affectively similar to real archery.

On the contrary, the participants reported greater perceived difficulty after real shooting than after VR shooting. In other words, individuals found real archery more challenging and taxing than its VR counterpart. This result, together with physiological data, clearly indicated that VR archery in its current form is not as demanding as real archery. After real archery, participants reported significantly higher levels of RPE compared to their reported RPE after VR archery. Research has indicated that individuals found VR archery to be simpler and experienced less‐perceived difficulty and exertion compared to real archery. This suggests that the physical accuracy of VR archery may be lower, meaning there is less resemblance between executing the task in VR and real life. Furthermore, the study found that participants achieved better shooting accuracy and accumulated more shooting points in VR archery than in real archery, providing further evidence for the possibility of lower fidelity in VR archery.

It is important to note that the current study has some limitations. First, the participants were all beginner‐level archers. Therefore, exploring the psychophysiological responses of experienced or elite archers during both VR and real archery may provide a more comprehensive understanding of the subject matter. Additionally, comparing the electromyographical activity of the muscles involved in arrow shooting could lead to a deeper insight into the topic. Individuals’ attitudes toward technology use might also be taken into consideration to better understand the topic.

## 5 Conclusions

On the basis of the present study we concluded that VR archery has the potential to induce ANS responses to a certain extent, but it does not stimulate the ANS to the same degree as real archery. This suggests that real archery caused stronger exercise‐induced sympathetic nerve activity than VR archery. On the other hand, VR archery led to a more pronounced decrease in RMSSD compared to real archery. Self‐reported data supported this conclusion. The primary reason for the reduced ANS excitation during VR archery is the use of joysticks to shoot via virtual bow. Although the motor and perceptual skills required for VR and real archery are similar, the equipment used for each type of archery is different. In VR archery, two joysticks are used instead of a real bow, and they do not require the same physical effort to draw a bowstring. Consequently, shooting arrows in VR is significantly easier than in real life, potentially explaining the lower HR acceleration observed in VR archery conditions. Our findings suggest that VR equipment used for athletic tasks should replicate real equipment to elicit psychophysiological responses similar to those in real life. The relatively lower HR elevation observed during VR archery indicates that it may not be effective in evoking sympathetic nerve activity, suggesting that the physical fidelity of VR archery is somewhat weak. Unlike VR archery, real archery promoted a vagal rebound effect, as evidenced by increased RMSSD. The increase in RMSSD during real archery and its decrease in VR archery may be attributed to the vagal rebound effect and vagal withdrawal, respectively, caused by the distinct patterns of sympathetic nerve activity in the two types of archery.

The present results might have several implications for both athletes and coaches in archery. Accordingly, VR archery applications have the potential to help beginners develop these skills and generate interest in the sport. Specifically, VR archery can be used in developing beginners’ fundamental archery skills, such as drawing the bowstring, correct posture, eye‐hand coordination, and aiming instead of rob exercise.

## Author Contributions

Research concept and study design: Nihal Dal, Serdar Tok, and Erdal Binboğa. Literature review: Serdar Tok, Erdal Binboğa, and Nihal Dal. Data collection: Erdal Binboğa, Nihal Dal, Said Enes Yılmaz, and İlker Balıkçı. Data analysis and interpretation: Serdar Tok, Erdal Binboğa, Nihal Dal, and Said Enes Yılmaz. Statistical analyses: Serdar Tok, Erdal Binboğa, and Nihal Dal. Writing of the manuscript: Serdar Tok, Erdal Binboğa, Nihal Dal, and Said Enes Yılmaz. Editing a draft of the manuscript: Serdar Tok, Erdal Binboğa, Nihal Dal, Said Enes Yılmaz, and İlker Balıkçı.

## Ethics Statement

This study's protocol was approved by the Manisa Celal Bayar University Institute of Health Sciences Ethics Committee, Manisa, Turkey with a reference number of 20.478.486/1423. All data were collected in accordance with the Declaration of Helsinki.

## Conflicts of Interest

The authors declare no conflicts of interest.

### Peer Review

The peer review history for this article is available at https://publons.com/publon/10.1002/brb3.70070.

## Data Availability

The datasets generated during and/or analyzed during the current study are available from the corresponding author upon reasonable request.
